# Quantitative imaging of intraerythrocytic hemozoin by transient absorption microscopy

**DOI:** 10.1117/1.JBO.25.1.014507

**Published:** 2019-12-17

**Authors:** Andy J. Chen, Kai-Chih Huang, Selina Bopp, Robert Summers, Puting Dong, Yimin Huang, Cheng Zong, Dyann Wirth, Ji-Xin Cheng

**Affiliations:** a Purdue University, Department of Biological Sciences, West Lafayette, Indiana, United States; b Boston University, Photonics Center, Boston, Massachusetts, United States; c Boston University, Department of Biomedical Engineering, Boston, Massachusetts, United States; d Harvard T.H. Chan School of Public Health, Boston, Massachusetts, United States; e Boston University, Department of Electrical and Computer Engineering, Boston, Massachusetts, United States; f Boston University, Department of Chemistry, Boston, Massachusetts, United States

**Keywords:** malaria, hemozoin, transient absorption microscopy, label-free imaging, chemical imaging

## Abstract

Hemozoin, the heme detoxification end product in malaria parasites during their growth in the red blood cells (RBCs), serves as an important marker for diagnosis and treatment target of malaria disease. However, the current method for hemozoin-targeted drug screening mainly relies on *in-vitro* β-hematin inhibition assays, which may lead to false-positive events due to under-representation of the real hemozoin crystal. Quantitative *in-situ* imaging of hemozoin is highly desired for high-throughput screening of antimalarial drugs and for elucidating the mechanisms of antimalarial drugs. We present transient absorption (TA) imaging as a high-speed single-cell analysis platform with chemical selectivity to hemozoin. We first demonstrated that TA microscopy is able to identify β-hematin, the artificial form of hemozoin, from the RBCs. We further utilized time-resolved TA imaging to *in situ* discern hemozoin from malaria-infected RBCs with optimized imaging conditions. Finally, we quantitatively analyzed the hemozoin amount in RBCs at different infection stages by single-shot TA imaging. These results highlight the potential of TA imaging for efficient antimalarial drug screening and drug mechanism investigation.

## 1 Introduction

Malaria, which is caused by the infection of *Plasmodium* species, infects over 200 million people and caused 430,000 deaths worldwide in 2017, according to the World Health Organization.^1^ Although there have been advances in prevention, fast diagnosis, and improved antimalarial treatment, artemisinin-resistant and multidrug-resistant *Plasmodium falciparum* strains pose a new threat to public health worldwide. Thus, methods for better understanding the drug mechanisms and efficient screening of innovative antimalarial compounds are highly desired.^2^^,^^3^

During the blood stage of malaria infection, the *Plasmodium* species utilizes hemoglobin as the main nutrient source.^4^ The breakdown of hemoglobin releases free heme, which is toxic to the malaria parasite.^5^ To detoxify free heme, the parasite catalyzes the polymerization of free heme and transforms it into insoluble hemozoin crystal.^5^ Hemozoin crystal, therefore, serves as the sink for minerals and heme.^6^ It was reported that small molecular drugs directly or indirectly inhibit the formation of hemozoin crystal and effectively kill malaria parasites.^7^ In addition, targeting hemozoin formation, which is specific to the parasite, reduces the concern of disrupting host pathways, such as nucleotide and protein synthesis machineries, by other drugs targeting purine and pyrimidine metabolic pathways of *Plasmodium*.^8^ Therefore, screening for chemicals targeting hemozoin formation is important for antimalarial drug development.^9^^,^^10^

Hemozoin-formation-inhibiting antimalarial drug development requires target-based high-throughput screening (HTS) and target pathway validation.^9^ Currently, the most prominent hemozoin-based HTS is *in-vitro* β-hematin inhibition assays.^9^ The β-hematin inhibition assay utilizes β-hematin, a synthetic analog of hemozoin, as a simple, robust, and cost-effective assay to screen antimalarial compounds. There are two main methods for early β-hematin inhibition assays: one is based on the detection of radioactive $C^{14}$-labeled hematin incorporated into the growing hemozoin crystal by using a scintillation counter.^11^ This method is sensitive but hazardous and expensive. The other method quantifies β-hematin via infrared light absorption.^12^ This method does not need radiolabeled hematin but is time consuming. It requires centrifugations and 48 h of sample drying before recording the infrared spectrum, which limits its application for HTS.^12^^,^^13^ After the initial round of *in-vitro* screening, positive hits are selected. However, not all the positive hits can inhibit hemozoin formation *in situ*. Therefore, high-throughput *in-situ* quantitative imaging of both hemozoin and intracellular heme is highly desired to confirm disruption of hemozoin formation as the drug target.^9^

Transient absorption (TA) microscopy is sensitive for mapping endogenous pigments or nonfluorescent molecules in a label-free manner.^14^^–^^17^ TA microscopy utilizes two synchronized pulsed beams, namely the pump and probe beams, to interrogate the excited state molecular dynamics. The pump beam first excites the electrons from ground state to an excited state, which can be detected by the following probe beam at different pump–probe temporal delays. TA microscopy uses time-zero TA intensity or time-resolved TA signal as an image contrast to differentiate chemical species and has been applied in various scientific fields, including material science, biology fields, and translational medicine studies.^18^^–^^22^ For biomedical applications, TA imaging is shown to be able to differentiate deoxyhemoglobin and oxyhemoglobin,^23^ discriminate melanomas,^19^ and quantify glycated hemoglobin for diabetes diagnosis.^24^ Moreover, as an absorption-based method, TA is more sensitive to small-sized particles, compared to the scattering-based methods.^25^ Therefore, we propose using TA microscopy as a quantitative and sensitive single-cell analysis platform to *in-situ* study intraerythrocytic hemozoin.

We first utilized our lab-built TA microscope to differentiate β-hematin from red blood cells (RBCs). In addition, we demonstrated that TA microscopy is able to *in situ* differentiate hemozoin crystal from hemoglobin within RBCs. Hemozoin crystal in infected RBCs can be detected as early as the ring stage. Finally, we quantitatively analyzed the size and number of hemozoin at various stages of malaria infection and observed an increase in both the crystal size and crystal number corresponding to the infected RBCs along with a decrease of TA signal from the RBCs. Our proof-of-concept study shows the potential of using a TA microscope for high-throughput *in-vitro* β-hematin inhibition assays and *in-situ* antimalarial drug target validation.

## 2 Materials and Methods

### 2.1 β-Hematin Synthesis

β-hematin was synthesized using an acid precipitation method documented previously.^26^ Specifically, nitrogen gas was constantly pumped into 0.1 M sodium hydroxide for 15 min to deoxygenize the solution. Then 50 mg hemin chloride (Sigma Aldrich 51280) was dissolved in 10 ml deoxygenized sodium hydroxide solution and was kept away from light. About 0.4 ml propionic acid was added drop by drop over a 20-min frame with the solution stirred at 200 rpm. The mixture was annealed for 18 h at 70°C. The amorphous aggregates were removed by washing with 0.1 M sodium bicarbonate alternating with water wash for three times. Then, the sample was washed with high performance liquid chromatography (HPLC) grade methanol (Sigma Aldrich 34860) alternating with water applied three more times. The solid products were dried over phosphorus pentoxide (Sigma Aldrich 431419) overnight to produce β-hematin crystal.

### 2.2 Scanning Electron Microscopy

Scanning electron microscopy (SEM) of β-hematin was performed on a Zeiss Supra 55 VP system. β-hematin crystal suspensions were dispersed in ethanol and later dried on a silicon wafer. The specimen was mounted on aluminum stubs and coated with platinum for SEM imaging at an electron beam voltage of 3 kV and a beam aperture of 30  μm.

### 2.3 Time-Resolved Transient Absorption Microscopy

The setup of a time-resolved TA microscope is shown in Fig. S1 in the Supplementary Material. Our lab-built TA imaging system used a femtosecond pulsed laser (Spectra-Physics Insight) operating at 80 MHz with two synchronized outputs. The pump beam was modulated at ∼2.5  MHz using an acoustic-optical modulator (Isomet 1205-C). The probe beam went through a delay-tuning stage and combined with the pump beam. The combined beams were directed to a 60×, 1.2 numerical aperture (NA) water immersing objective (Olympus UPlanApo/IR). After passing through the specimen, the transmitted probe beam was collected using a 1.4-NA oil condenser (Olympus U-AAC), filtered with a bandpass filter, and detected by a photodiode (Hamamatsu 3994). The modulated TA signal was first amplified by a lab-built resonant circuit and then demodulated by a lock-in amplifier (Zurich Instrument HF2LI) with a time constant set to 7  μs. To record the time-resolved TA signal, a delay-tuning stage was applied to tune the temporal delay between the pump pulse and the probe pulse with 66.7 fs per step. The pump–probe delay is scanned in a frame-by-frame manner to generate a three-dimensional data cube (x−y−τ). A 200×200×120 (x−y−τ) data cube requires 48 s to acquire with a pixel dwell time set to 10  μs. No photodamage was observed for RBC after data acquisition. Data were processed using ImageJ and analyzed using Origin software.

### 2.4 Spontaneous Raman Spectroscopy

Spontaneous Raman spectroscopy of synthesized β-hematin was performed on a Horiba confocal Raman microscope (Horiba Scientific, Labram HR Evolution). A continuous wave laser at 785 nm was used for excitation. The laser was focused by a 40× air objective with a laser power of 35 mW on the sample. Each Raman spectral acquisition was accumulated for 10 s. The pinhole size was set to 50  μm to reject the out-of-focus background. A 600-grooves/mm grating was utilized to disperse the light and generate a Raman spectrum. Crystals of β-hematin were screened through transmission imaging to determine the point of interest for Raman spectroscopy.

### 2.5 *P. falciparum* Culture and Sample Preparation

*P. falciparum* 3D7 strain was cultured by standard methods.^27^ The parasites were kept in cell culture flasks (Corning 430825) containing blood (New York blood center) and Roswell Park Memorial Institute (RPMI) 1640 medium (Thermo Fisher 11875093) supplemented with 28 mM ${\mathrm{NaHCO}}_{3}$, 25 mM N-2-hydroxyethylpiperazine-N'-2-ethanesulfonic acid (HEPES) buffer solution, 25  μg/mL gentamicin, and 0.5% AlbuMAX II (Life Technologies 11021-045). The culture flasks were kept in a cell culture incubator at 37°C. Culture medium was changed on a daily basis. *P. falciparum* 3D7 was diluted about twice per week to keep the parasitemia below 5%. During subculture, *P. falciparum* culture was synchronized using sorbitol.^28^ The culture was then maintained as described above. *P. falciparum* fixation is done by immersing in 100% methanol (Sigma Aldrich 322415) for 4 s.

### 2.6 Spectral Phasor Analysis

Time-resolved TA signals at each pixel were mapped into a two-dimensional (2-D) phasor space with the coordinates g and s, which are the real and imaginary parts of the time-resolved TA signal after Fourier transformation, respectively.^24^^,^^29^ The coordinates g and s can be expressed as g(ω)=∫I(t)×cos(ωt)dt/∫|I(t)|dt, and s(ω)=∫I(t)×sin(ωt)dt/∫|I(t)|dt, where I(t) is the time-resolved TA signal and ω is the given frequency, which is set to 1.0 here. Clusters of hemozoin and RBCs in the phasor plot were separated manually, as shown in Fig. 3(c). Spectral phasor analysis was performed using an ImageJ plugin software.^30^ To quantitatively analyze the TA decay rates of each cluster, the retrieved TA decay curves were approximated by fitting with a biexponential decay function as shown below: $y=A1\times \exp(-t/\tau_{1})+A2\times \exp(-t/\tau_{2})+y0$, where t is the pump–probe delay, A1 is the amplitude, and $\tau_{i}$ are the decay time constants. The fast decay time constant $\tau_{1}$ was fixed at 0.38 ps as the system response time determined by the laser pulse duration. The TA decay rate was retrieved by fitting the slow decay time constant $\tau_{2}$.

## 3 Results

### 3.1 Time-Resolved Transient Absorption Imaging to Differentiate β-Hematin and Heme in Red Blood Cells

TA microscopy is shown to be able to map heme and heme-related products, such as imaging of heme dynamics in *Caenorhabditis elegans*^22^ and detection of glycated hemoglobin (HbA1c) in single RBCs.^24^ Since hemozoin is synthesized from the polymerization of heme dimer,^31^ we tested whether a time-resolved TA signal can be used to differentiate the artificial hemozoin, β-hematin, from the heme in RBC.

#### 3.1.1 Characterization of β-hematin

**Fig. 1:**
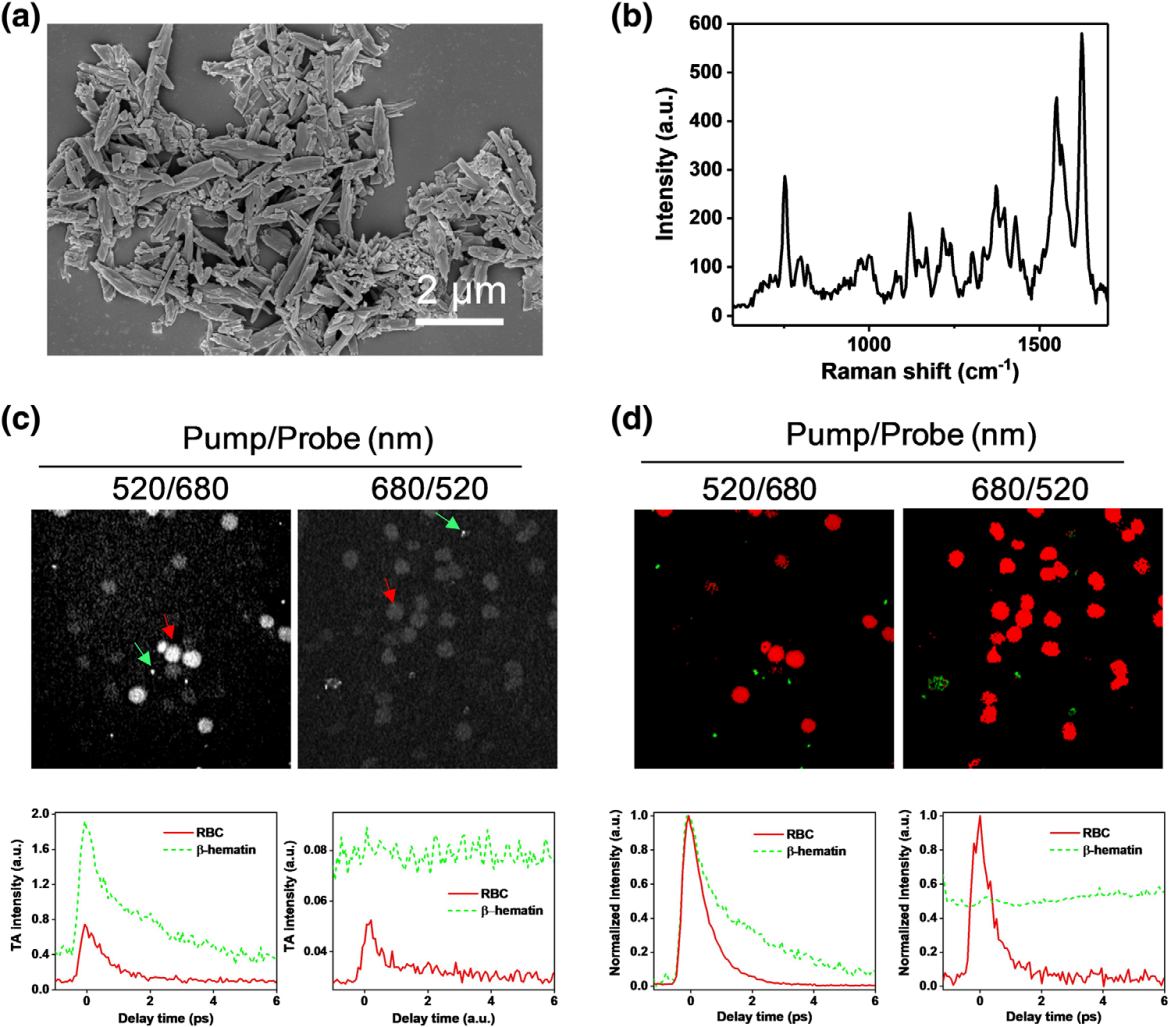
TA microscopy spectroscopically differentiates β-hematin from hemoglobin in RBCs. (a) SEM image of synthesized β-hematin. (b) Raman spectrum of β-hematin: laser wavelength: 785 nm; laser power: 35 mW on the sample; dwell time: 10 s; objective: 40× air; pinhole size: 50  μm, grating: 600  l/mm. (c) Time-resolved pump–probe images of β-hematin and RBC mixture. Left panel: pump/probe=520/680  nm; right panel: pump/probe=680/520  nm; all images are at zero-time delay, dynamic range: 0.25 to 0.73; graphs are the decay curves of the β-hematin and RBCs indicated by the arrows (green and red, respectively); laser power: 10 mW for each beam; pixel dwell time: 10  μs; 60× water objective. (d) Phasor analysis outputs of the images in (c). (c)–(d) Left panel: pump/probe=520/680  nm; right panel: pump/probe=680/520  nm. (c)–(d) Top panel: retrieved images from phasor analysis (β-hematin is artificially colored green and RBCs in red); bottom panel: graphs are retrieved pump–probe spectra from phasor analysis.

We synthesized β-hematin from hemin chloride using an acid precipitation protocol.^26^ SEM confirmed the synthesized β-hematin with the desired flake shape and with a size ranging from 0.5 to 2.0  μm, similar to natural hemozoin [Fig. 1(a)].^32^ Spontaneous Raman scattering spectroscopy was performed to confirm the chemical composition. As shown in Fig. 1(b), the characteristic peak at $750\;{\mathrm{cm}}^{-1}$, which is assigned to the full pyrrole ring breathing modes $\nu_{15}$, indicates the β-hematin structure in the synthesized crystals.^33^^,^^34^ Notably, the peak pattern from 1300 to $1600\;{\mathrm{cm}}^{-1}$ resembles the Raman spectrum of hemin [Fig. 1(b)],^33^ indicating the remnant of hemin. However, the hemin remnant, which is likely in a powder format, did not confuse the experimental result later because individual β-hematin crystals were mixed with RBCs instead of hemin.

#### 3.1.2 Transient absorption signal of β-hematin and its dependence on excitation laser wavelength

Having acquired β-hematin with high quality, we next performed time-resolved TA imaging on the mixture of β-hematin and RBCs. First, we performed a pump and probe wavelength dependence experiment to optimize the TA signal of β-hematin with either a 520/680  nm or a 680/520  nm pump/probe combination. From the images at zero pump–probe temporal delay, we observed that β-hematin has substantially higher TA intensity than RBCs in both imaging conditions [Fig. 1(c), top panel]. This observation was consistent with the decay curve generated from the image stacks, in which the intensities of β-hematin were about twice that of the RBCs [Fig. 1(c), bottom panel]. We also noted that β-hematin and RBCs produced very different pump–probe decay curves, which can be used as a spectroscopic contrast. Therefore, we applied spectral phasor analysis to project the TA decay curve from each pixel onto a 2-D phasor domain.^29^ We observed distinctive distribution patterns of RBCs and β-hematin from the background on the phasor plots (see Fig. S2 in the Supplementary Material). Notably, the β-hematin crystals, originally with weak TA intensities acquired under the 680/520  nm pump/probe combination, were apparent in the phasor map. We further normalized the retrieved decay curves and compared the decay curve as shown in the bottom panel of Fig. 1(d). We observed that when the pump beam was set to 520 nm, the β-hematin showed distinctively slower decay dynamics, with a decay time constant of 2.47±0.16  ps, than RBCs, with a decay time constant of 0.52±0.01  ps [Fig. 1(d), bottom left panel). When the pump beam was set to 680 nm, the pump–probe decay curve of β-hematin suggests that the signal is mainly contributed from the thermal effect due to the signal insensitivity to the pump–probe decay position, whereas RBCs retained an identical asymmetry TA decay curve [Fig. 1(d), bottom right panel]. In either case, the pump–probe decay patterns could serve as a spectroscopic signature to differentiate β-hematin from RBCs.

### 3.2 Time-Resolved Transient Absorption Imaging of Hemozoin within Infected Red Blood Cells

#### 3.2.1 Transient absorption signal of hemozoin and its dependence on excitation laser wavelength

**Fig. 2:**
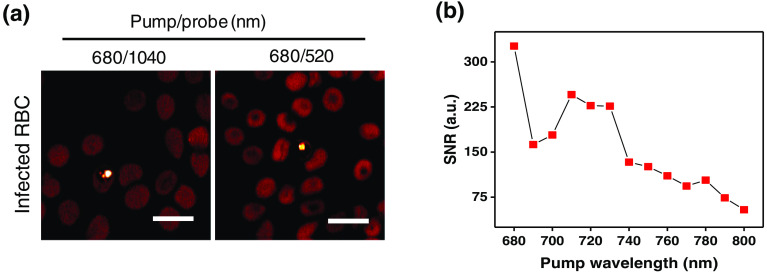
Hemozoin TA signal dependence on excitation laser wavelength. (a) TA images of hemozoin using 680/1040 and 680/520  nm as the pump/probe wavelengths, respectively. Sample: RBCs infected with *P. falciparum* 3D7 strain; laser power: 10 mW for each beam; scale bar: 25  μm; pixel dwell time: 10  μs. Left panel: pump/probe=680/1040  nm; dynamic range: 0.1 to 1.8; right panel: pump/probe=680/520  nm; dynamic range: 0.1 to 0.7. (b) SNR of TA signal using different pump wavelengths. Sample was a single hemozoin crystal from RBCs infected with *P. falciparum* 3D7. Probe beam wavelength: 1040 nm; laser power: 10 mW for each beam, pixel dwell time: 10  μs; SNR was calculated by first determining the intensity difference of a fixed area of hemozoin and an equal area of the RBC, then dividing the intensity difference by the standard deviation of the RBC.

Having proved that TA signals could separate β-hematin from RBCs, we next tested whether TA imaging could identify natural hemozoin within infected RBCs. We first optimized the hemozoin imaging condition with different pump–probe laser wavelength combinations. The optimization was performed under the constraint of our laser with one tunable beam ranging from 680 to 1300 nm and the other beam fixed at 1040 nm or 520 nm through frequency doubling. Since the absorbance difference of hemozoin and hemoglobin is largest at 680 nm,^35^ we hypothesize that hemozoin gives a stronger TA signal when pumped at 680 nm compared with that at 520 or 1040 nm.^36^ Indeed, we found that the 680/520  nm pump/probe combination obtained a higher signal-to-noise ratio (SNR) than the reversed combination (see Fig. S3 in the Supplementary Material). Later, we optimized the probe wavelength with the pump beam set to 680 nm. From the representative images, 1040-nm probing resulted in higher hemozoin signal normalized with the signals from the RBC [Fig. 2(a)], indicating that 1040 nm is a better probe wavelength. Finally, to confirm that 680 nm was the optimal pump wavelength, we carried out a pump wavelength dependency study. We fixed the probe beam at 1040 nm and compared SNRs generated from pump wavelengths ranging from 680 to 800 nm, with 10 nm increments [Fig. 2(b)]. The result shows that a pump wavelength of 680 nm optimizes the SNR. We conclude that the 680/1040  nm pump/probe combination is the optimal condition for *in-situ* TA imaging of hemozoin.

#### 3.2.2 Time-resolved transient absorption imaging to identify natural hemozoin in 48-h infected red blood cells

**Fig. 3:**
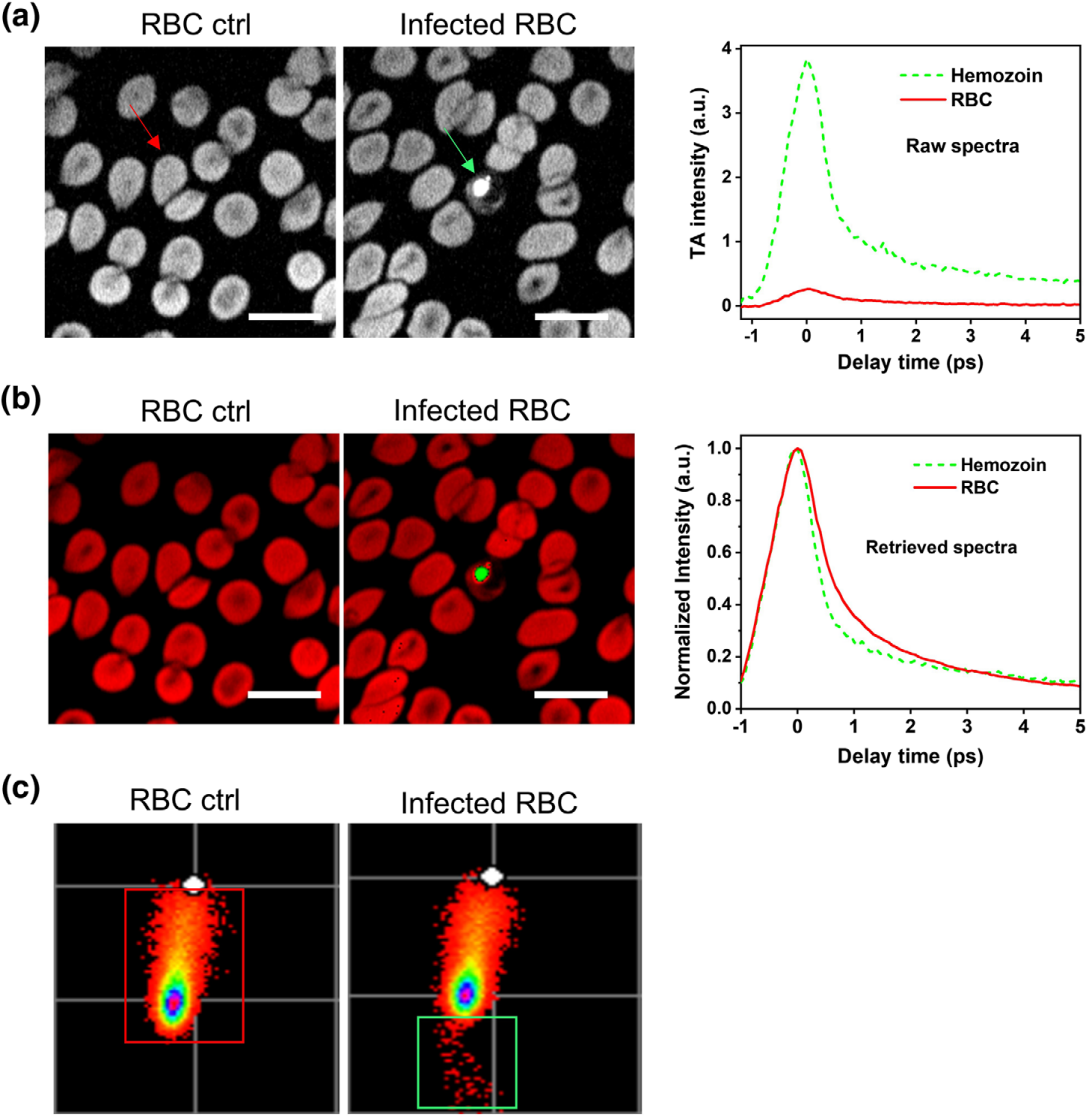
Time-resolved TA imaging and phasor analysis differentiates hemozoin from hemoglobin in RBCs. (a) Raw TA images and decay curves of malaria-infected RBCs. Sample: RBCs infected with *P. falciparum*; pump/probe=680/1040  nm; laser power: 10 mW for each beam. Left panel: images at zero-time delay; scale bar: 25  μm; image display range: 0.02 to 0.5; RBC and hemozoin selected for generating decay curves were indicated with arrows. Right panel: raw TA decay curves of RBC and hemozoin; an area of about 100 pixels from both indicated objects were selected to plot the decay curves. The curves were smoothed by averaging among five nearby data points. (b) Mapping result retrieved from phasor analysis of data in (a). Left panel: retrieved images; red: RBC; green: hemozoin; scale bar: 25  μm. Right panel: retrieved decay curves of hemozoin and RBCs; intensity was normalized to 1.0. (c) Phasor plot retrieved from phasor analysis of data in (a). Dots within red box: RBCs; dots within green box: hemozoin.

With the optimized imaging condition, we then tested whether time-resolved TA imaging is able to differentiate hemozoin from hemoglobin in RBCs infected with late-stage *P. falciparum*. From the TA images at zero-time pump–probe delay, intracellular hemozoin crystal is clearly identified with a higher TA intensity [Fig. 3(a), left panel]. By plotting the pump–probe decay curves from the hemoglobin region of RBCs and hemozoin, which is indicated by the arrow in the left panel of Fig. 3(a), we found both showing TA decay patterns, but hemozoin differed substantially from RBC with around nine times larger TA intensity [Fig. 3(a), right panel]. We further applied phasor analysis to generate maps of hemozoin and RBC based on their TA decay rates [Fig. 3(b), left panel]. In the phasor plot from the uninfected RBCs dataset, we observed only one RBC main group, as shown in the red rectangle of Fig. 3(c). In the phasor plot from infected RBCs, the hemozoin group located lower than the main RBCs group was marked with a green rectangle [Fig. 3(c)]. The retrieved spectra revealed that the hemozoin crystal decayed faster than hemoglobin, with the TA decay time constants of 1.22±0.12 and 1.70±0.11  ps, respectively [Fig. 3(b), right panel]. We observed that the hemozoin mapping result is consistent with the higher TA intensity region in the zero-time pump–probe delay TA image [Fig. 3(b), left panel]. Collectively, these data demonstrated that even single-shot TA at zero temporal delay is able to *in situ* identify hemozoin from RBCs in late stages of malarial infection. In the next section, we used this simplified and faster approach for large area mapping of intracellular hemozoin in different infection stages.

### 3.3 *In Situ* Quantitation of Hemozoin within Different Infection Stages of Red Blood Cells

**Fig. 4:**
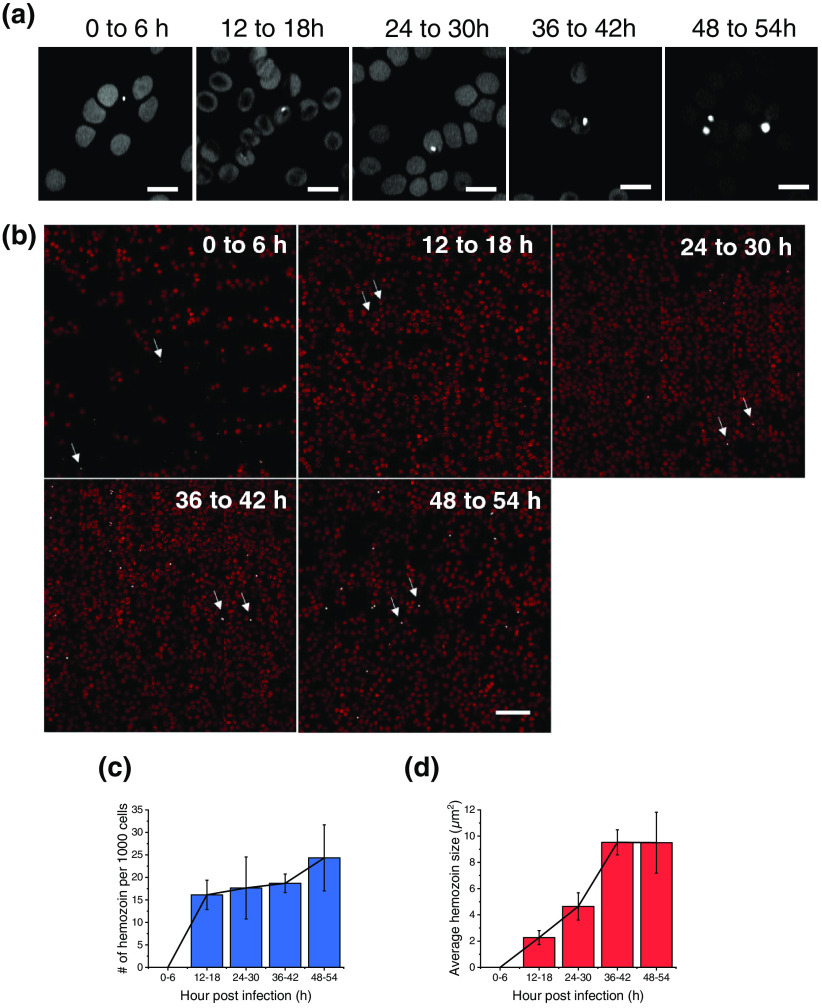
Quantitative analysis of hemozoin in RBCs at different stages of malarial infection. (a) Representative single-shot TA images of infected RBCs. Sample: RBCs infected with *P. falciparum* 3D7 0, 12, 24, 36, and 48 h after invasion; since individual *P. falciparum* differs during synchronization and infection process, there is a range of 6 h at the beginning of synchronization, which expands to 12 h starting from 48 h after synchronization. Consequently, the time points were denoted as 0 to 6, 12 to 18, 24 to 30, 36 to 42 and 48 to 54 h; image was taken at zero-time delay; pump/probe: 680/1040  nm; laser power: 30 mW for each beam; scale bar: 20  μm; pixel dwell time: 10  μs; (b) 7×7 large area mapping of samples in (a). Images were artificially colored red; representative hemozoin crystals were indicated by arrows; scale bar: 100  μm; displaying ranges were adjusted to standardize the RBCs: 0 to 6 h = 0.03 to 0.23, 12 to 18 h = 0.02 to 0.2, 24 to 30 h = 0.01 to 0.34, 36 to 42 h = 0.03 to 0.48, 48 to 54 h = 0.03 to 0.23. (c) The graph of the number of hemozoin crystals at different time points after infection; crystal number was normalized to 1000 RBCs. (d) The graph of the size of hemozoin crystal at different time points after infection. Hemozoin crystal size was determined by applying a threshold to filter out RBC background and then the total number of pixels of the hemozoin is converted into square micrometers.

Having demonstrated the capability of a TA microscopy to *in situ* differentiate hemozoin from hemoglobin, we further explored how early in the malarial infection stage TA imaging could detect hemozoin. To this end, we tightly synchronized parasites (6-h window) and made fixed smears at 0, 12, 24, 36, and 48 h postinfection. We found that in the 0 h of infection, no intracellular hemozoin was detected. Instead, only small individual hemozoin crystals dispersing in the medium were observed [Fig. 4(a), 0 to 6 h]. These extracellular crystals were likely the remnants from the synchronization process.^28^ We observed intracellular hemozoin crystal for cells after 12 h infection [Fig. 4(a), 2 to 18 h]. As infection proceeded, both crystal size and number increased [Fig. 4(a)]. Notably, contrary to the hemozoin, for which the TA signal increased with infection time, the hemoglobin signal continuously decreased as the infection progressed [Fig. 4(a)]. This observation demonstrates the power of TA imaging to visualize both hemoglobin degradation and hemozoin accumulation over the 48-h lifecycle of *P. falciparum.*

We further performed large area mapping of thousands of RBCs in order to quantitatively analyze the growth of hemozoin in the rarely infected RBCs. With the estimated seeding parasitemia as 0.1%, and at least one hemozoin crystal produced by each parasite, we decided to sample at least 1000 cells to capture the rare event of hemozoin crystal formation in each image. Given that each single frame covers about 25 cells on average, a 7×7 large area mapping covers more than 1000 cells. The 0- to 6-h sample contains fewer than 1000 cells due to low cell density, but this does not affect the quantification result because there was no intracellular hemozoin observed [Fig. 4(b)]. However, 12 h after infection, hemozoin could be detected and quantified [Fig. 4(b)]. Since the number of infected RBCs did change over the next 48 h, the number of hemozoin crystals only increased slightly over the course of infection [Fig. 4(c)]. In contrast, the crystal size increased substantially over time, reflecting the growth of hemozoin in each infected cell during mid-infection and late infection stages [Fig. 4(d)]. After 36 h, the average crystal size reached a plateau. Meanwhile, the number of hemozoin crystals started to increase again at 48 h, indicating the maturation and rupture of the first-generation *P. falciparum* and the invasion of new RBCs by the second generation [Fig. 4(c)]. These data collectively show the potential of using TA microscopy to quantitate intracellular hemozoin growth in size and number over a full lifecycle of *P. falciparum* infection.

## 4 Discussion

Because of the emergence and fast evolution of multidrug-resistant malaria strains, efficient and HTS for alternative antimalarial drugs is highly desired.^2^ Validation of the positive hits and a thorough understanding of drug mechanisms are essential to achieve this goal.^9^ Here, we demonstrate TA microscopy as a sensitive, label-free, and chemical-selective method that enables *in-situ* quantitative detection of heme components in the forms of hemozoin crystals and hemoglobin in RBCs. We first synthesized β-hematin crystal and showed that TA imaging is sensitive to detect β-hematin crystal by either TA intensity or time-resolved TA signal. We determined the optimal pump–probe wavelength combination as 680/1040  nm. *In-situ* quantitative study of hemozoin and RBCs in different infection stages was demonstrated. TA imaging is found to be able to detect hemozoin as early as after 12 h post infection. The TA signal of hemozoin increased while the signal of hemoglobin decreased as the infection progressed. Thousands of RBCs were screened to quantitatively monitor the change in the size and number of hemozoin *in situ* over a complete lifecycle of *P. falciparum*. These proof-of-concept data show the potential of using TA imaging for high-throughput antimalarial drug screening by monitoring hemozoin formation *in situ*. Our method overcomes the limitation of *in-vitro* β-hematin inhibition assay in its under-representation of the real hemozoin crystal, which leads to a large number of false positives while missing potential valuable hits.^9^ Our current system can be further upgraded to an imaging flow cytometry setting by conjugating with a hydrodynamic focusing system to further improve the throughput.

TA microscopy, as a sensitive tool for hemozoin, can be combined with coherent Raman scattering microscopy to monitor drug dynamics in infected RBCs. Visualization of the interaction between drugs and hemozoin will provide valuable insights to elucidate drug mechanisms. For example, it is known that quinoline suppresses *P. falciparum* by inhibiting hemozoin synthesis. However, the drug mechanism is still in debate as to whether it is through direct sequestration of heme^36^ or through binding to the growing face of hemozoin.^37^ Such mysteries can be unveiled by the combination of TA microscopy with coherent Raman scattering microscopy, which is a label-free chemical imaging tool with the capability of imaging small molecules.^38^^,^^39^ Such a multimodal imaging platform is expected to provide critical insights into the working mechanisms of antimalarial drugs.
