# Supplementary material for: Quantitative imaging of intraerythrocytic hemozoin by transient absorption microscopy
Source: J Biomed Opt. 2019 Dec 17;25(1):014507. doi: 10.1117/1.JBO.25.1.014507 (PMC6916744; doi:10.1117/1.JBO.25.1.014507)
Supplement: Supplementary file 1 [file JBO_025_014507_SD001.docx]

**Quantitative imaging of** **intra-erythrocytic hemozoin by** **transient absorption microscopy**

Andy J. Chen^a*^, Kai-Chih Huang^b, c*^, Selina Bopp^d^, Robert Summers^d^, Pu-Ting Dong^b^, Yimin Huang^b^, Cheng Zong^b^, Dyann Wirth^d^, Ji-Xin Cheng^b,c,e,f,*^

aDepartment of Biological Sciences, Purdue University, West Lafayette, IN. 47907

bPhotonics Center, Boston University, Boston, MA. 02215

cDepartment of Biomedical Engineering, Boston University, Boston, MA. 02215

dHarvard T.H. Chan School of Public Health, Boston, MA. 02115

eDepartment of Electrical & Computer Engineering, Boston University, Boston, MA. 02215

fDepartment of Chemistry, Boston University, Boston, MA. 02215

*****Corresponding Author**,** E-mail: [jxcheng@bu.edu](mailto:jxcheng@bu.edu)

**Supporting figures**

**
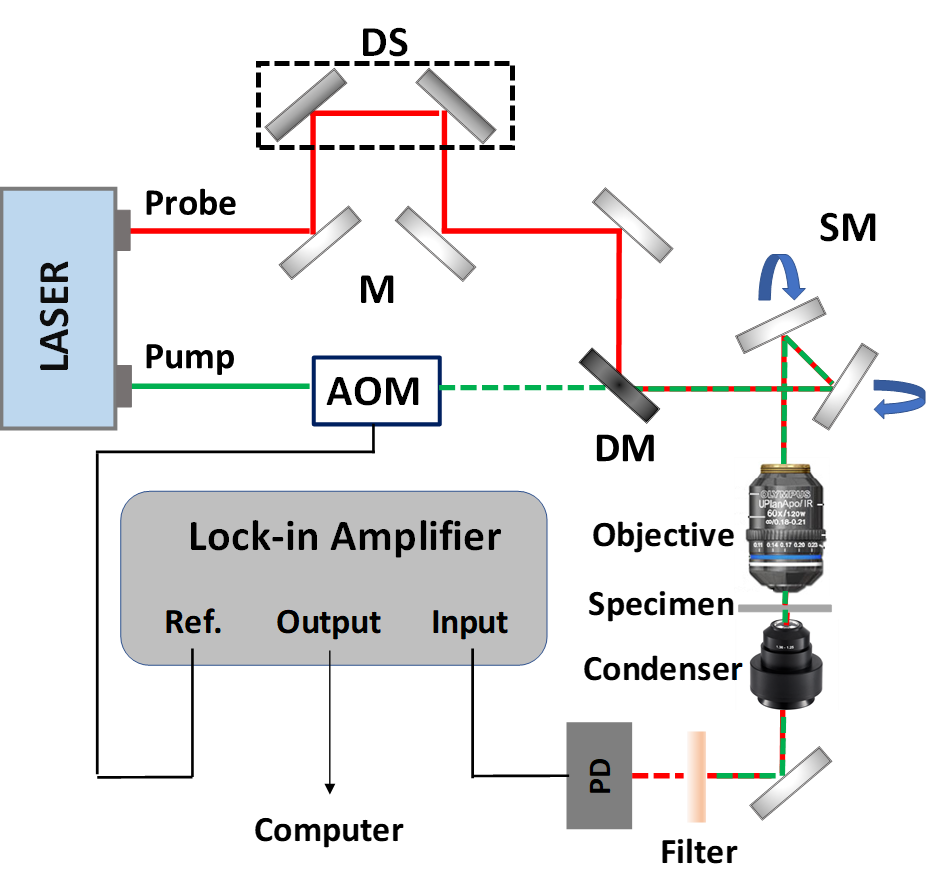
**

**Figure S1 Schematic of TA microscope.**

A femtosecond laser source provides two synchronized outputs, a fixed wavelength pump beam and a tunable probe beam. The Stokes beam is modulated by an AOM. The pump beam is combined with the probe beam by a dichroic mirror. A motorized delay stage is installed on the probe beam to scan the temporal delay between pump and probe. The combined lasers are scanned by a pair of scanning mirrors and focused by an objective length. After collecting the transmission light by a condenser, a filter is used to filter out the pump beam. The TA signal on the probe beam is detected by a photodiode, and extracted by a lock-in amplifier. DS, delay stage. M, mirror. DM, dichroic mirror. SM, scanning mirrors. AOM, acousto-optic modulator. PD, photodiode.

**Figure S2 Phasor plot of β-hematin and red blood cells.**

a. Phasor plots of β-hematin and red blood cells using 520 and 680 nm as pump and probe beam, respectively. Selected dots were corresponding to the component, the rest dots were from background. b. Same data analysis and display, with pump and probe wavelengths switched.

**Figure S3 Single-shot transient absorption imaging separates β-hematin from RBCs.**

The transient absorption images of hematin and RBCs are acquired with pump/probe wavelength of 520/680 nm for the left image and of 680/520 nm for the right image. Laser power: 10 mW for each beam; scale bar: 100 μm; pixel dwell time: 10 μs; objective: 40X; images were taken at zero-time-delay.
